# Supplementary material for: Questionnaire-free machine-learning method to predict depressive symptoms among community-dwelling older adults
Source: PLoS One. 2023 Jan 25;18(1):e0280330. doi: 10.1371/journal.pone.0280330 (PMC9876369; doi:10.1371/journal.pone.0280330)
Supplement: S1 File — This file consists of: (1) S1 Table. Guidelines for developing and reporting machine learning predictive models in biomedical research; (2) S2 Table. Prediction model risk of bias assessment tools (PROBAST); (3) S3 Table. Clinical checklists for assessing suitability of machine learning applications in healthcare; and (4) S4 Table. The 15-item Geriatric Depression Scale (GDS-15) questionnaire. (DOCX) [file pone.0280330.s001.docx]

# Table S1. Guidelines for developing and reporting machine learning predictive models in biomedical research

| **Item number** | **Section** | **Topic** | **Checklist item with the description and/or the corresponding text** | | |
| --- | --- | --- | --- | --- | --- |
| 1 | Title | Nature of study | ✓ |  | Identify the report as introducing a predictive model |
|  |  |  |  | • | Questionnaire-free machine-learning method to predict depressive symptoms among community-dwelling older adults |
| 2 | Abstract | Structured summary | ✓ |  | Background |
|  |  |  |  | • | The 15-item Geriatric Depression Scale (GDS-15) is widely used to screen for depressive symptoms among older populations. |
|  |  |  | ✓ |  | Objectives |
|  |  |  |  | • | This study aimed to develop and validate a questionnaire-free, machine learning model as an alternative triage test for the GDS-15 among community-dwelling older adults. |
|  |  |  | ✓ |  | Data sources |
|  |  |  |  | • | Data collected from 15 community health centers in Indonesia by a cross-sectional design were utilized (*n*=1381). |
|  |  |  | ✓ |  | Performance metrics of the predictive model or models, in both point estimates and confidence intervals |
|  |  |  |  | • | The AUROC of the RF model was 0.619 (95% CI 0.610 to 0.627) for the external validation set with a non-local ethnic group. |
|  |  |  | ✓ |  | Conclusion including the practical value of the developed predictive model or models |
|  |  |  |  | • | Our triage test can allow healthcare professionals to preliminarily screen for depressive symptoms in older adults without use of a questionnaire. If the model shows positive results, then the GDS-15 can be used for follow-up measures. This will save a lot of time and energy for both healthcare providers and older adults especially those persons who are illiterate. |
| 3 | Introduction | Rationale | ✓ |  | Identify the clinical goal |
|  |  |  |  | • | A triage test with questionnaire-free variables is needed to reduce the frequency of questionnaire use. |
|  |  |  | ✓ |  | Review the current practice and prediction accuracy of any existing models |
|  |  |  |  | • | Almost all existing predictive models of depressive symptoms include questionnaire-based predictors, e.g., the Patient Health Questionnaire (PHQ), the Edinburgh Postnatal Depression Scale (EPDS), and the GDS. |
|  |  |  |  | • | Several questionnaire-based screening tools are still needed for more-frequent identification of patients with depression (with an area under the receiver operating characteristic [ROC] curve [AUROC] of 0.700, 95% confidence interval [CI] 0.629 to 0.771), which were a part of the Self-Reported Quick Inventory of Depressive Symptomatology (QIDS-SR) and Hamilton Depression Rating Scale (HAM-D). |
|  |  |  |  | • | An extended predictD algorithm was developed to predict major depression 12~24 months later based on the DSM-IV (AUROC 0.728, 95% CI 0.675 to 0.781; *n*=2670), but this also needs a subject to fill in the 12-Item Short Form (SF-12) for two of the predictors. |
|  |  |  |  | • | One study utilized a wearable device to predict GDS-15 and HAM-D results in older adults (AUROC 0.96, 95% CI 0.91 to 0.99; *n*=47); unfortunately, the sample size was very small, and a wearable device might not be affordable for some older adults. |
|  |  | Objectives | ✓ |  | State the nature of study being predictive modelling, defining the target of prediction |
|  |  |  |  | • | This study aimed to develop and validate a questionnaire-free model to predict the GDS-15 among community-dwelling older adults by machine learning. |
|  |  |  | ✓ |  | Identify how the prediction problem may benefit the clinical goal |
|  |  |  |  | • | These factors may be utilized to develop a prediction model as a triage test for the GDS-15 at any time, while the screening frequency of GDS-15 can be reduced by restricting respondents to only those who test positive according to the prediction model. |
| 5 | Methods | Describe the setting | ✓ |  | Identify the clinical setting for the target predictive model |
|  |  |  |  | ❖ | Clinical setting: primary care |
|  |  |  |  |  |  |
|  |  |  |  | • | On June to September 2019, this dataset was collected using a cross-sectional design from 15 community health centers (CHCs) in Kendari, Indonesia (*n*=1381). |
|  |  |  | ✓ |  | Identify the modelling context in terms of facility type, size, volume, and duration of available data |
|  |  |  |  | ❖ | Facility type: community health centre (CHC) |
|  |  |  |  | ❖ | Size: 1,381 older adults |
|  |  |  |  | ❖ | Volume: 15 CHCs |
|  |  |  |  | ❖ | Duration: 4 months (June to September 2019) |
|  |  |  |  |  |  |
|  |  |  |  | • | On June to September 2019, this dataset was collected using a cross-sectional design from 15 community health centers (CHCs) in Kendari, Indonesia (*n*=1381). All patients aged 60 years or older with clear consciousness who visited the CHCs were enrolled. |
| 6 | Methods | Define the prediction problem | ✓ |  | Define a measurement for the prediction goal (per patient or per hospitalization or per type of outcome) |
|  |  |  |  | ❖ | Prediction goal: positive result of the GDS per participant |
|  |  |  |  |  |  |
|  |  |  |  | • | The event definition for this prediction task was depressive symptoms based on the GDS-15. |
|  |  |  | ✓ |  | Determine that the study is retrospective or prospective |
|  |  |  |  | ❖ | Study design: previously collected dataset by cross-sectional design |
|  |  |  |  |  |  |
|  |  |  |  | • | We utilized a dataset collected from our previous project investigating loneliness and depression in older adults. On June to September 2019, this dataset was collected using a cross-sectional design from 15 community health centers (CHCs) in Kendari, Indonesia (*n*=1381). |
|  |  |  | ✓ |  | Identify the problem to be prognostic or diagnostic |
|  |  |  |  | ❖ | Prediction problem: diagnostic |
|  |  |  |  |  |  |
|  |  |  |  | • | The event definition for this prediction task was depressive symptoms based on the GDS-15. |
|  |  |  | ✓ |  | Determine the form of the prediction model: ((KPCPEN), #3219) classification if the target variable is categorical, (2) regression if the target variable is continuous, (3) survival prediction if the target variable is the time to an event. |
|  |  |  |  | ❖ | Prediction model form: classification |
|  |  |  |  |  |  |
|  |  |  |  | • | The event definition for this prediction task was depressive symptoms based on the GDS-15. |
|  |  |  | – |  | Translate survival prediction into a regression problem, with the target measured over a temporal window following the time of prediction. |
|  |  |  |  | ❖ | Not applicable |
|  |  |  | ✓ |  | Explain practical costs of prediction errors (e.g., implications of under-diagnosis or over-diagnosis) |
|  |  |  |  | ❖ | Under-diagnosis: failed prevention of major depressive disorders |
|  |  |  |  | ❖ | Over-diagnosis: higher frequency of GDS-15 but considerably non-trivial implication |
|  |  |  |  |  |  |
|  |  |  |  | • | Under-diagnosis causes missed cases of depressive symptoms when screened using the GDS-15, which leads to failure to prevent major depressive disorders. Meanwhile, over-diagnosis causes an increasing frequency of the use of the GDS-15, which may lead to further misclassification. Nonetheless, the risk of under-diagnosis outweighs the that of over-diagnosis. |
|  |  |  | ✓ |  | Defining quality metrics for prediction models |
|  |  |  |  | ❖ | Quality metrics: calibration metric and AUROC |
|  |  |  |  |  |  |
|  |  |  |  | • | We used the area under receiver operating characteristics (ROC) curve (AUROC) as the main evaluation metric. This is because the AUROC is threshold-agnostic. |
|  |  |  |  | • | But, before evaluating this, we reported the calibration metric of a model using an LR in which the predicted probability as the model output became the only covariate. The models were considered well calibrated if the 95% CIs of the intercept and slope respectively covered 0 and 1, with the probability plots visually aligned with the reference line. |
|  |  |  | ✓ |  | Define the success criteria for prediction (e.g., based on metrics in internal validation or external validation in the context of the clinical problem) |
|  |  |  |  | ❖ | Success criteria: the best, well-calibrated model that is robust based on external validation |
|  |  |  |  |  |  |
|  |  |  |  | • | We chose all models that complied with the calibration metric. |
|  |  |  |  | • | The best models were well-calibrated models that significantly out-performed others according to the AUROC. All metrics are reported with the 95% CI. A model outperformed the others if the interval estimate was greater than the central value of the other models. Otherwise, more than one model might be selected. |
|  |  |  |  | • | The best model was determined using the internal validation set, and should be robust based on all external validation sets, and for which the central value of the AUROC was approximately >0.5. |
| 7 | Methods | Prepare data for model building | ✓ |  | Identify relevant data sources and quote the ethics approval number for data access |
|  |  |  |  | ❖ | Data source: secondary dataset |
|  |  |  |  | ❖ | Ethical clearance was waived. One for collecting the original data: TMU Joint Institutional Review Board (approval number: N201905105) and The Ethical Research Committee in Universitas Halu Oleo (approval number: 954/UN.29.20/PPM/2018) |
|  |  |  |  |  |  |
|  |  |  |  | • | We utilized a dataset collected from our previous project investigating loneliness and depression in older adults. |
|  |  |  |  | • | Ethical clearance for this study was waived by Taipei Medical University (TMU). The original study was granted ethical clearance by the TMU Joint Institutional Review Board (approval no.: N201905105) and The Ethical Research Committee in Universitas Halu Oleo (approval no.: 954/UN.29.20/PPM/2018). |
|  |  |  | ✓ |  | State the inclusion and exclusion criteria for data |
|  |  |  |  | ❖ | Inclusion criteria: patient of CHC aged 60 years or older |
|  |  |  |  | ❖ | Exclusion criteria: - |
|  |  |  |  |  |  |
|  |  |  |  | • | All patients aged 60 years or older with clear consciousness who visited the CHCs were enrolled. |
|  |  |  | ✓ |  | Describe the time span of data and the sample or cohort size |
|  |  |  |  | ❖ | Time span of data: June to September 2019 |
|  |  |  |  | ❖ | Sample size: 1,381 older adults |
|  |  |  |  |  |  |
|  |  |  |  | • | On June to September 2019, this dataset was collected using a cross-sectional design from 15 community health centers (CHCs) in Kendari, Indonesia (*n*=1381). |
|  |  |  | ✓ |  | Define the observational units on which the response variable and predictor variables are defined |
|  |  |  |  | ❖ | Response variable for classification task: depression status positive or negative by score of >5 based on GDS-15 |
|  |  |  |  | ❖ | Predictors: 1) age; 2) gender; 3) religion; 4) education; 5) marital status; 6) children; 7) living status; 8) currently employed; 9) previously employed; 10) income; 11) visit CHC; 12) comorbidity; 13) health condition; 14) hearing problem; 15) visual problem; 16) oral status; 17) medication. |
|  |  |  |  |  |  |
|  |  |  |  | • | The dataset consisted of 19 attributes: 1) age (years); 2) gender (male/female); 3) religious beliefs (Christian/Hindu/Moslem); 4) educational attainment (illiterate/primary/secondary/high school/university/other); 5) marital status (single/married/separated or divorced/widowed); 6) children (number of persons); 7) living status (alone/with a family member but no spouse/with a spouse only/with family member and spouse/other); 8) currently employed (no/yes); 9) previously employed (no/yes); 10) income (in Indonesian rupiah (IDR)); 11) duration of visiting the CHC (in the number of years of routine visits); 12) comorbidities (number of conditions); 13) health condition (very good/good/fair/poor/very poor); 14) hearing problems (no/yes); 15) visual problems (no/yes); 16) oral status (very good/good/fair/poor/very poor); 17) medication (number of prescribed drugs); 18) ethnicity (Bugis-Makassar/Buton/Muna/Tolaki/non-local ethnicity); and 19) depressive symptoms (no/yes). The first 17 attributes were used as predictors. |
|  |  |  |  | • | As the predicted outcome, depressive symptoms were assessed based on the GDS. There were 15 questions to obtain a score (which ranged 0 to 15). Some items give a point if answered positively while others give a point if answered negatively. If the total score is greater than 5, the scale suggests that a person has depressive symptoms. |
|  |  |  | ✓ |  | Define the predictor variables. Extra caution is needed to prevent information leakage from the response variable to predictor variables. |
|  |  |  |  | ❖ | Numerical predictors: 1) age (year); 2) children (number of persons); 3) income (IDR); 4) visit CHC (duration in year of routine visits); 5) comorbidity (number of conditions); 6) medication (number of prescribed drug). |
|  |  |  |  | ❖ | Categorical predictors (after binarisation into no = 0 or yes = 1): 7) male gender; 8) female gender; 9) Christian religion; 10) Moslem religion; 11) illiterate; 12) primary education; 13) secondary education; 14) high school; 15) university; 16) other education; 17) single; 18) married; 19) separated or divorced; 20) widowed; 21) living alone; 22) living with family member but no spouse; 23) living with spouse only; 24) living with family member and spouse; 25) currently employed; 26) previously employed; 27) very good health condition; 28) good health condition; 29) fair health condition; 30) poor health condition; 31) very poor health condition; 32) hearing problem; 33) visual problem; 34) very good oral status; 35) good oral status; 36) fair oral status; 37) poor oral status. |
|  |  |  |  |  |  |
|  |  |  |  | • | The first 17 attributes were used as predictors. |
|  |  |  |  | • | The GDS questionnaire is described in in Table S4. |
|  |  |  |  | • | We only used data partitioning for model development to conduct predictor extraction, representation, and selection. For candidate predictors, the binarized predictors were extracted only for those without a perfect separation problem in which the predictor was found in only one of the outcomes. |
|  |  |  |  | • | Of 40 predictors after binarization, only 37 were extracted. The excluded predictors were living status of "other", oral status of "very poor", and religion of "Hindu". |
|  |  |  | ✓ |  | Describe the data pre-processing performed, including data cleaning and transformation. Remove outliers with impossible or extreme responses; state any criteria used for outlier removal. |
|  |  |  |  | ❖ | Data cleaning: remove variables with perfect separation in training set |
|  |  |  |  | ❖ | Data transformation: binarization of categorical predictors, standardisation and normalisation of numerical predictors |
|  |  |  |  | ❖ | Outlier removal: limit numerical predictors within 95% confidence interval after standardization |
|  |  |  |  |  |  |
|  |  |  |  | • | All categorical predictors were binarized into 0 or 1 for "no" or "yes" as to whether a category applied to a participant. |
|  |  |  |  | • | All numerical predictors were standardized using the mean and standard deviation (SD) but capped at the 2.5% and 97.5% quantiles as the respective minimum and maximum values. This resulted in a value range of approximately -1.96 to 1.96. |
|  |  |  |  | • | We only used data partitioning for model development to conduct predictor extraction, representation, and selection. For candidate predictors, the binarized predictors were extracted only for those without a perfect separation problem in which the predictor was found in only one of the outcomes. |
|  |  |  |  | • | Of 40 predictors after binarization, only 37 were extracted. The excluded predictors were living status of "other", oral status of "very poor", and religion of "Hindu". |
|  |  |  | ✓ |  | State how missing values were handled |
|  |  |  |  | ❖ | Missing value handling: multiple imputation by chain equation in each subset |
|  |  |  |  |  |  |
|  |  |  |  | • | We checked for missing values in the dataset. The only missing value was found in visual problems for one participant (*n*=1/1381, 0.072%). This was missing completely at random since we got this information from routine physical health check data. We imputed the missing value using multiple imputation by the chain equation method after data transformation using only data in the same data partition. |
|  |  |  | ✓ |  | Describe the basic statistics of the dataset, particularly of the response variable. These include the ratio of positive to negative classes for a classification problem and the distribution of the response variable for regression problem. |
|  |  |  |  | ❖ | Classification outcome ratio: training set (609 [+]:393 [-]), random external validation set (153 [+]:97 [-]), non-random external validation set (67 [+]:62 [-]) |
|  |  |  |  |  |  |
|  |  |  |  | • | Table 1. |
|  |  |  | ✓ |  | Define the model validation strategies. Internal validation is the minimum requirement; external validation should also be performed whenever possible. |
|  |  |  |  | ❖ | External validation: non-random external validation from non-local ethnicity, random external validation from ~20% of any local ethnicity |
|  |  |  |  |  |  |
|  |  |  |  | • | We used participants with ethnicity not from Sulawesi Island for the external validation set. This may demonstrate the model robustness to predict outcomes in the general population. We also randomly split the remaining set after excluding the external validation set. This provided another external validation set with as much as ~20% of the remaining set. |
|  |  |  | ✓ |  | Specify the internal validation strategy. Common methods include random split, time-based split, and patient-based split. |
|  |  |  |  | ❖ | Internal validation: 30-time bootstrapping (model training, comparison, and re-calibration), 10-fold cross validation (hyperparameter tuning and principal component analysis), 20% hold-out cross validation (DI-VNN training iteration) |
|  |  |  |  |  |  |
|  |  |  |  | • | For the first to third models, we applied 10-fold cross-validation for hyperparameter tuning and 30 times bootstrapping for training the model using the best hyperparameters. We also applied 10-fold cross-validation to compute the rotated matrix of PCs. For the fourth model, we applied a hold-out cross-validation with 80:20 ratios for the training and validation sets. To compare this model against the others, we applied 30 times bootstrapping to compute the predictive performance. To re-calibrate all models using a logistic regression, we also applied 30 times bootstrapping. |
|  |  |  | ✓ |  | Define the validation metrics. For regression problems, the normalized root-mean-square error should be used. For classification problems, the metrics should include sensitivity, specificity, positive predictive value, negative predictive value, area under the ROC curve, and calibration plot. |
|  |  |  |  | ❖ | Validation metrics: area under the ROC curve and calibration metric and plot |
|  |  |  |  |  |  |
|  |  |  |  | • | We used the area under receiver operating characteristics (ROC) curve (AUROC) as the main evaluation metric. This is because the AUROC is threshold-agnostic. |
|  |  |  |  | • | But, before evaluating this, we reported the calibration metric of a model using an LR in which the predicted probability as the model output became the only covariate. The models were considered well calibrated if the 95% CIs of the intercept and slope respectively covered 0 and 1, with the probability plots visually aligned with the reference line. |
|  |  |  | ✓ |  | For retrospective studies, split the data into a derivation set and a validation set. For prospective studies, define the starting time for validation data collection. |
|  |  |  |  | ❖ | Data partition: non-random external validation from non-local ethnicity, random external validation from ~20% of any local ethnicity |
|  |  |  |  |  |  |
|  |  |  |  | • | We used participants with ethnicity not from Sulawesi Island for the external validation set. This may demonstrate the model robustness to predict outcomes in the general population. We also randomly split the remaining set after excluding the external validation set. This provided another external validation set with as much as ~20% of the remaining set. |
| 8 | Methods | Build the predictive model | ✓ |  | Identify independent variables that predominantly take a single value (e.g., being zero 99% of the time) |
|  |  |  |  | ❖ | Number of independent variables with zero variance: 3 predictors |
|  |  |  |  |  |  |
|  |  |  |  | • | We only used data partitioning for model development to conduct predictor extraction, representation, and selection. For candidate predictors, the binarized predictors were extracted only for those without a perfect separation problem in which the predictor was found in only one of the outcomes. |
|  |  |  |  | • | Of 40 predictors after binarization, only 37 were extracted. The excluded predictors were living status of "other", oral status of "very poor", and religion of "Hindu". |
|  |  |  | ✓ |  | Identify and remove redundant independent variables |
|  |  |  |  | ❖ | Number of redundant independent variables: 0 |
|  |  |  |  |  |  |
|  |  |  |  | • | We assessed redundant predictors assisted by Pearson's correlation coefficients. Two binarized predictors were highly correlated (*r*=0.72), which were "living with family members without a spouse" and a "widowed" marital status. We decided to retain these variables because the correlation was near borderline and was apparently due to sampling bias. A "widowed" marital status is not necessarily living with family members. An older adult might live alone. This was also considerably not to be interchangeable. |
|  |  |  | ✓ |  | Identify the independent variables that may suffer from the perfect separation problem |
|  |  |  |  | ❖ | Perfect-separation variables: living status of other, oral status of very poor, and religion of Hindu |
|  |  |  |  |  |  |
|  |  |  |  | • | We only used data partitioning for model development to conduct predictor extraction, representation, and selection. For candidate predictors, the binarized predictors were extracted only for those without a perfect separation problem in which the predictor was found in only one of the outcomes. |
|  |  |  |  | • | Of 40 predictors after binarization, only 37 were extracted. The excluded predictors were living status of "other", oral status of "very poor", and religion of "Hindu". |
|  |  |  | ✓ |  | Report the number of independent variables, the number of positive examples, and the number of negative examples |
|  |  |  |  | ❖ | Number of independent variables: 19 PCs for LR, 7 PCs for SPC-RF and SPC-GBM, and 18 candidate features for DI-VNN with 609 [+] and 393 [-] in training set |
|  |  |  |  |  |  |
|  |  |  |  | • | Of 40 predictors after binarization, only 37 were extracted. The excluded predictors were living status of "other", oral status of "very poor", and religion of "Hindu". |
|  |  |  |  | • | To optimize the predictive performance, we applied a dimension-reduction technique using a principal component (PC) analysis (PCA). We only used the top 19 PCs based on the percent variance explained because we needed to comply with the sample size for predictive modeling based on PROBAST guidelines, which is 20 events per variable or candidate of predictors (see "Model Validation"). A ten-fold cross-validation procedure was applied. We used average values computed from ten rotated matrices of PCs to represent 37 binarized and numerical predictors into 19 PCs. We also used average values of data partitioning for model development to get those PCs for model validation. |
|  |  |  |  | • | We also used other machine learning algorithms beside the logistic regression to develop prediction models (see "Model Development"). However, the models required larger sample sizes which were >50 events per variable. We used the wrapper method in which we selected PCs using a logistic regression before being candidate predictors for the machine learning models. We applied the same hyperparameter tuning strategy of the LR for this predictor selection (see "Model Development"). |
|  |  |  |  | • | Some modifications of this pipeline were those by applying this procedure over 37 predictors and 19 PCs resulting 18 candidate features for DI-VNN. These were centred using each average value after quantile-to-quantile normalization over all features among samples. |
|  |  |  |  | • | Table 1. |
|  |  |  | ✓ |  | Assess whether sufficient data are available for a good fit of the model. In particular, for classification, there should be a sufficient number of observations in both positive and negative classes. |
|  |  |  |  | ❖ | Classification problem: 20 EPV for LR, 56 EPV for SPC-RF and SPC-GBM, and 21 EPV for DI-VNN |
|  |  |  |  |  |  |
|  |  |  |  | • | Of 40 predictors after binarization, only 37 were extracted. |
|  |  |  |  | • | We only used the top 19 PCs based on the percent variance explained because we needed to comply with the sample size for predictive modeling based on PROBAST guidelines,58 which is 20 events per variable or candidate of predictors (see "Model Validation"). |
|  |  |  |  | • | However, the models required larger sample sizes which were >50 events per variable. We used the wrapper method in which we selected PCs using a logistic regression before being candidate predictors for the machine learning models. |
|  |  |  |  | • | Some modifications of this pipeline were those by applying this procedure over 37 predictors and 19 PCs resulting 18 candidate features for DI-VNN. |
|  |  |  |  | • | Table 1. |
|  |  |  | ✓ |  | Determine a set of candidate modelling techniques (e.g., logistic regression, random forest, or deep learning). If only one type of model was used, justify the decision for using that model. |
|  |  |  |  | ❖ | Candidate modelling techniques: LR, SPC-RF, SPC-GBM, and DI-VNN |
|  |  |  |  |  |  |
|  |  |  |  | • | We developed four models with different approaches. First, we applied the simplest model using a logistic regression (LR) with a shrinkage method as recommended by the PROBAST guidelines. |
|  |  |  |  | • | The second and third prediction models were developed using RF and gradient boosting machine (GBM) algorithms. |
|  |  |  |  | • | We used the wrapper method in which we selected PCs using a logistic regression before being candidate predictors for the machine learning models. |
|  |  |  |  | • | The last prediction model was developed using the deep-insight visible neural network (DI-VNN) algorithm. |
|  |  |  | ✓ |  | Define the performance metrics to select the best model |
|  |  |  |  | ❖ | Performance metrics for model selection: AUROC interval estimates |
|  |  |  |  |  |  |
|  |  |  |  | • | The best models were well-calibrated models that significantly out-performed others according to the AUROC. |
|  |  |  | ✓ |  | Specify the model selection strategy. Common methods include K-fold validation or bootstrap to estimate the lost function on a grid of candidate parameter values. For K-fold validation, proper stratification by the response variable is needed. |
|  |  |  |  | ❖ | Model selection strategy: highest interval estimate of AUROC by bootstrapping |
|  |  |  |  |  |  |
|  |  |  |  | • | To compare this model against the others, we applied 30 times bootstrapping to compute the predictive performance. |
|  |  |  |  | • | All metrics are reported with the 95% CI. A model outperformed the others if the interval estimate was greater than the central value of the other models. Otherwise, more than one model might be selected. The best model was determined using the internal validation set, and should be robust based on all external validation sets, and for which the central value of the AUROC was approximately >0.5. |
|  |  |  | ✓ |  | (A desirable but not mandatory item) For model selection, include discussion on ((KPCPEN), #3219) balance between model accuracy and model simplicity or interpretability, and (2) the familiarity with the modelling techniques of the end user |
|  |  |  |  | ❖ | Modelling balance between accuracy and interpretability: LR is more interpretable, SPC-RF and SPC-GBM are more accurate, and DI-VNN has a moderate performance and deeper interpretability. |
|  |  |  |  | ❖ | Familiarity with the modelling techniques: LR is a well-known statistical model, SPC-RF and SPC-GBM is a state-of-the-art machine learning algorithm, and DI-VNN is a pipeline of well-known genomic analysis applied to deal high-dimensionality problem, along with a well-known CNN deep learning algorithm modified for deeper interpretability. |
|  |  |  |  |  |  |
|  |  |  |  | • | We developed four models with different approaches. First, we applied the simplest model using a logistic regression (LR) with a shrinkage method as recommended by the PROBAST guidelines. |
|  |  |  |  | • | The second and third prediction models were developed using RF and gradient boosting machine (GBM) algorithms. Both are state-of-the-art algorithms that were shown to consistently outperform other algorithms across different outcomes. |
|  |  |  |  | • | Both algorithms are the most used competition-winning algorithms for predictions using tabular data. |
|  |  |  |  | • | The last prediction model was developed using the deep-insight visible neural network (DI-VNN) algorithm. This is a deep-learning model or a convolutional neural network (CNN). This model emerged in recent years because it improves predictive performance for imaging data. The Deep Insight algorithm converts a non-image into image-like data as a multidimensional array in a meaningful way using a dimensional-reduction algorithm over the predictors. The VNN means that the network architecture is data-driven, because it is determined based on a hierarchical clustering algorithm over the predictors. This addresses criticisms of the CNN as a black-box model for which characteristics do not imply the data but predict the outcome very well. |
| 9 | Results | Report the final model and performance | ✓ |  | Report the predictive performance of the final model in terms of the validation metrics specified in the methods section |
|  |  |  |  | ❖ | Predictive performance report: See Table 2 |
|  |  |  |  |  |  |
|  |  |  |  | • | Table 2. |
|  |  |  | ✓ |  | If possible, report the parameter estimates in the model and their confidence intervals. When the direct calculation of confidence intervals is not possible, report nonparametric estimates from bootstrap samples. |
|  |  |  |  | ❖ | Parameter estimates: See Figure 2 and 3 |
|  |  |  |  |  |  |
|  |  |  |  | • | Figure 2. |
|  |  |  |  | • | Figure 3. |
|  |  |  | ✓ |  | Comparison with other models in the literature should be based on confidence intervals |
|  |  |  |  | ❖ | Other models in the literature: a previous model using a wearable device to predict GDS-15 in older adults living alone (*n*=47) |
|  |  |  |  |  |  |
|  |  |  |  | • | In addition, a previous study also applied a questionnaire-free method to predict the GDS-15 in older adults living alone using a wearable device, but the model was considerably overfit because of a very small sample size (AUROC 0.96, 95% CI 0.91 to 0.99; *n*=47). |
|  |  |  | ✓ |  | Interpretation of the final model. If possible, report what variables were shown to be predictive of the response variable. State which subpopulation has the best prediction and which subpopulation is most difficult to predict. |
|  |  |  |  | ❖ | Predictive variables: See Figure 2 |
|  |  |  |  | ❖ | Subpopulation with the best prediction: older adults aged 60 or older who routinely visit CHCs, have 2 to 6 children, are mostly not separated/divorced, live with spouse and/or other family members in majority, are unemployed and low income, and are religious believers. |
|  |  |  |  | ❖ | Subpopulation that was most difficult to predict: older adults who have a high education, are single, have previous employment, have jobs, and do not have religion |
|  |  |  |  |  |  |
|  |  |  |  | • | Future use of our models will likely benefit those with similar characteristics, particularly in the predictors used in the final model. As intended, our models were developed for older adults aged ≥60 years. This characterizes older adults as reasonably having comorbidities and poorer health conditions of hearing, oral status, and visual function, which are considerable compared to those of younger adults. However, in all of those categorical variables (excluding comorbidities), the majority were in a fair health condition, probably because these subjects had routinely visited a CHC for up to 9 or 10 years on average. Most of the subjects had not obtained a university education. They had two to six children, were mostly not separated/divorced, and the majority lived with a spouse and/or other family members, although they were unemployed and their incomes were considerably low for this country. Most of the subjects, if not all, were religious believers. We saw similar characteristics between GDS-15 positives and negatives, except for the Tolaki (p=0.012) and Bugis-Makasar ethnic groups (p=0.48), the number of comorbidities (p=0.005), the employment status before 60 years of age (p=0.002), male gender (p=0.035), a poor health condition (p=0.016), a living alone status (p=0.011), and a separated/divorced status (p=0.042). |
|  |  |  |  | • | Figure 2. |
| 10 | Discussion | Clinical implications | ✓ |  | Report the clinical implications derived from the obtained predictive performance. For example, report the dollar amount that could be saved with better prediction. How many patients could benefit from a care model leveraging the model prediction? And to what extent? |
|  |  |  |  | ❖ | Potential cost efficiency: reduced unnecessary costs for the related diagnostic procedure and intervention caused by physical manifestation of later-life depression |
|  |  |  |  | ❖ | Potential healthcare impact: detection of depression under 1 year (monthly basis) without increasing measurement error of GDS-15 due to response fatigue |
|  |  |  |  |  |  |
|  |  |  |  | • | One can use these models in our web application to screen for depressive symptoms along with the GDS-15 at any time. If deemed to be positive according our models, an older adult is only then asked to answer questions in the GDS-15. This allows for more-frequent screening and may help detect depressive symptoms at any time. Since later-life depression often causes multiple physical symptoms, we would expect reduced unnecessary costs for related diagnostic procedures and interventions. |
| 11 | Discussion | Limitations of the model | ✓ |  | Discuss the following potential limitations: • Assumed input and output data format; • (Desirable but not mandatory items) Potential pitfalls in interpreting the model; • Potential bias of the data used in modelling; • Generalizability of the data |
|  |  |  |  | ❖ | Assumed input and output data format: religion and income input may not be well-determined |
|  |  |  |  | ❖ | Potential pitfalls in interpreting the model: different religion and different notion of income in any country may differently affect depressive symptoms |
|  |  |  |  | ❖ | Potential bias of the data used in modelling: similar characteristics of an older adult to the population is needed for optimal prediction |
|  |  |  |  | ❖ | Generalizability of the data: SPC-GBM is undifferentiated with DI-VNN |
|  |  |  |  |  |  |
|  |  |  |  | • | An older adult who is an atheist or believes in a religion beyond those in our dataset might not be well-predicted. The Big Mac index considerably perceives income as a notion of primary need, which is food, while depressing problems related to income may manifest as different notions. Populations with similar characteristics to those in our training set are warranted to use our prediction models. The predictive performance may differ if older adults have a high education, are single, have previous employment, have a job, and have no religious beliefs. More-similar characteristics to our target population would lead to more-optimal predictive performance. Although the SPC-GBM with re-calibration had the best performance in the internal validation set among the well-calibrated models, the performances were undifferentiated in the external validation set with local ethnicity compared to the DI-VNN without re-calibration. Nonetheless, we only used the internal validation set to choose the best model. This is because choosing the best model by the external validation set might lead to an optimistic bias or overfitting; instead, external validation sets were used for a robustness test of the performance of the prediction models. |
| 12 | Discussion |  | ✓ |  | (Desirable but not mandatory items) Report unexpected signs of coefficients, indicating collinearity or complex interaction between predictor variables |
|  |  |  |  | ❖ | Unexpected signs of coefficients: there was opposite effect by education, but this is not comparable because of slightly difference scope of the predictor (literacy vs. education) |
|  |  |  |  |  |  |
|  |  |  |  | • | In PC11, older adults with low education but literate and living alone were strong predictors of GDS-15 positives. However, our findings contradicted previous findings that reported education was negatively correlated with depressive symptoms. |

# Table S2. Prediction model risk of bias assessment tools (PROBAST)

| **DOMAIN 1: Participants** | | | | | |
| --- | --- | --- | --- | --- | --- |
| **Risk of Bias** | | | | | |
| *Describe the sources of data and criteria for participant selection:* | | | | | |
|  | • | We utilized a dataset collected from our previous project investigating loneliness and depression in older adults. On June to September 2019, this dataset was collected using a cross-sectional design from 15 community health centers (CHCs) in Kendari, Indonesia (*n*=1381). | | | |
|  | • | All patients aged 60 years or older with clear consciousness who visited the CHCs were enrolled. | | | |
|  | • | We used participants with ethnicity not from Sulawesi Island for the external validation set. This may demonstrate the model robustness to predict outcomes in the general population. We also randomly split the remaining set after excluding the external validation set. This provided another external validation set with as much as ~20% of the remaining set. | | | |
|  | |  | | **Dev** | **Val** |
| 1.1 Were appropriate data sources used, e.g. cohort, RCT or nested case-control study data? | | | | Y | Y |
| 1.2 Were all inclusions and exclusions of participants appropriate? | | | | Y | Y |
| **Risk of bias introduced by selection of participants** | | | **RISK:** | Low | Low |
|  |  |  | *(low/ high/ unclear)* |  |  |
| *Rationale of bias rating:* | | | | | |
|  | ❖ | Cross-sectional design is clearly warranted for diagnostic prediction. | | | |
|  | ❖ | The selection criteria mimics real-world setting for implementation of the prediction model. | | | |
|  |  |  |  |  |  |
| **DOMAIN 2: Predictors** | | | | | |
| **Risk of Bias** | | | | | |
| *List and describe predictors included in the final model, e.g. definition and timing of assessment:* | | | | | |
|  | • | The dataset consisted of 19 attributes: 1) age (years); 2) gender (male/female); 3) religious beliefs (Christian/Hindu/Moslem); 4) educational attainment (illiterate/primary/secondary/high school/university/other); 5) marital status (single/married/separated or divorced/widowed); 6) children (number of persons); 7) living status (alone/with a family member but no spouse/with a spouse only/with family member and spouse/other); 8) currently employed (no/yes); 9) previously employed (no/yes); 10) income (in Indonesian rupiah (IDR)); 11) duration of visiting the CHC (in the number of years of routine visits); 12) comorbidities (number of conditions); 13) health condition (very good/good/fair/poor/very poor); 14) hearing problems (no/yes); 15) visual problems (no/yes); 16) oral status (very good/good/fair/poor/very poor); 17) medication (number of prescribed drugs); 18) ethnicity (Bugis-Makassar/Buton/Muna/Tolaki/non-local ethnicity); and 19) depressive symptoms (no/yes). The first 17 attributes were used as predictors. | | | |
|  | • | The trained assessor that assisted a participant to fill out the GDS questionnaire was also blinded to the predictor information. Predictor data were demographic data and routine physical health check results from other healthcare givers without knowing the assessment results of depressive symptoms. | | | |
|  | |  | | **Dev** | **Val** |
| 2.1 Were predictors defined and assessed in a similar way for all participants? | | | | PY | PY |
| 2.2 Were predictor assessments made without knowledge of outcome data? | | | | Y | Y |
| 2.3 Are all predictors available at the time the model is intended to be used? | | | | Y | Y |
| **Risk of bias introduced by predictors or their assessment** | | | **RISK:** | Low | Low |
|  |  |  | *(low/ high/ unclear)* |  |  |
| *Rationale of bias rating:* | | | | | |
|  | ❖ | Predictor assessment was obviously determined. | | | |
|  | ❖ | Predictor and outcome were measured by different personnel. | | | |
|  | ❖ | In this diagnostic study, all predictors were already available at the same time with the outcome. | | | |
|  |  |  |  |  |  |
| **DOMAIN 3: Outcome** | | | | | |
| **Risk of Bias** | | | | | |
| *Describe the outcome, how it was defined and determined, and the time interval between predictor assessment and outcome determination:* | | | | | |
|  | • | The dataset consisted of 19 attributes: 1) age (years); 2) gender (male/female); 3) religious beliefs (Christian/Hindu/Moslem); 4) educational attainment (illiterate/primary/secondary/high school/university/other); 5) marital status (single/married/separated or divorced/widowed); 6) children (number of persons); 7) living status (alone/with a family member but no spouse/with a spouse only/with family member and spouse/other); 8) currently employed (no/yes); 9) previously employed (no/yes); 10) income (in Indonesian rupiah (IDR)); 11) duration of visiting the CHC (in the number of years of routine visits); 12) comorbidities (number of conditions); 13) health condition (very good/good/fair/poor/very poor); 14) hearing problems (no/yes); 15) visual problems (no/yes); 16) oral status (very good/good/fair/poor/very poor); 17) medication (number of prescribed drugs); 18) ethnicity (Bugis-Makassar/Buton/Muna/Tolaki/non-local ethnicity); and 19) depressive symptoms (no/yes). The first 17 attributes were used as predictors. | | | |
|  | • | As the predicted outcome, depressive symptoms were assessed based on the GDS. There were 15 questions to obtain a score (which ranged 0 to 15). Some items give a point if answered positively while others give a point if answered negatively. If the total score is greater than 5, the scale suggests that a person has depressive symptoms. | | | |
|  | • | The GDS questionnaire is described in Table S4. The trained assessor that assisted a participant to fill out the GDS questionnaire was also blinded to the predictor information. Predictor data were demographic data and routine physical health check results from other healthcare givers without knowing the assessment results of depressive symptoms. | | | |
|  | |  | | **Dev** | **Val** |
| 3.1 Was the outcome determined appropriately? | | | | Y | Y |
| 3.2 Was a pre-specified or standard outcome definition used? | | | | Y | Y |
| 3.3 Were predictors excluded from the outcome definition? | | | | Y | Y |
| 3.4 Was the outcome defined and determined in a similar way for all participants? | | | | PY | PY |
| 3.5 Was the outcome determined without knowledge of predictor information? | | | | Y | Y |
| 3.6 Was the time interval between predictor assessment and outcome determination appropriate? | | | | Y | Y |
| **Risk of bias introduced by the outcome or its determination** | | | **RISK:** | Low | Low |
|  |  |  | *(low/ high/ unclear)* |  |  |
| *Rationale of bias rating:* | | | | | |
|  | ❖ | The goal is to predict GDS-15 in order to reduce the screening frequency and avoid response fatigue. Thus, the outcome is determined appropriately. | | | |
|  | ❖ | The outcome was assessed using a standard definition. | | | |
|  | ❖ | No predictor information was found in the GDS-15 questions. | | | |
|  | ❖ | All outcome was determined similarly using the same questionnaire and assisted by trained enumerators without knowing the predictors. | | | |
|  | ❖ | The predictors and outcome were assessed at the same visit. | | | |
|  |  |  |  |  |  |
| **DOMAIN 4: Analysis** | | | | | |
| **Risk of Bias** | | | | | |
| *Describe numbers of participants, number of candidate predictors, outcome events and events per candidate predictor:* | | | | | |
|  | • | (No censoring, competing risk, and sampling of controls) | | | |
|  | • | All categorical predictors were binarized into 0 or 1 for "no" or "yes" as to whether a category applied to a participant. All numerical predictors were standardized using the mean and standard deviation (SD) but capped at the 2.5% and 97.5% quantiles as the respective minimum and maximum values. This resulted in a value range of approximately -1.96 to 1.96. Then, we applied normalization by shifting the central value, which was zero, to 0.5 and scaling down the range by half; thus, the numerical predictors were within a range of 0 to 1. | | | |
|  | • | Of 40 predictors after binarization, only 37 were extracted. The excluded predictors were living status of "other", oral status of "very poor", and religion of "Hindu". | | | |
|  | • | We only used the top 19 PCs based on the percent variance explained because we needed to comply with the sample size for predictive modeling based on PROBAST guidelines, which is 20 events per variable or candidate of predictors (see "Model Validation"). | | | |
|  | • | However, the models required larger sample sizes which were >50 events per variable. We used the wrapper method in which we selected PCs using a logistic regression before being candidate predictors for the machine learning models. | | | |
|  | • | Table 1 and Table 2. | | | |
|  | • | Some modifications of this pipeline were those by applying this procedure over 37 predictors and 19 PCs resulting 18 candidate features for DI-VNN. These were centred using each average value after quantile-to-quantile normalization over all features among samples. | | | |
| *Describe how the model was developed (e.g. in regards to modelling technique (e.g. survival or logistic modelling), predictor selection, and risk group definition):* | | | | | |
|  | • | As the predicted outcome, depressive symptoms were assessed based on the GDS. | | | |
|  | • | We only used the top 19 PCs based on the percent variance explained because we needed to comply with the sample size for predictive modeling based on PROBAST guidelines, which is 20 events per variable or candidate of predictors (see "Model Validation"). | | | |
|  | • | However, the models required larger sample sizes which were >50 events per variable. We used the wrapper method in which we selected PCs using a logistic regression before being candidate predictors for the machine learning models. | | | |
|  | • | Some modifications of this pipeline were those by applying this procedure over 37 predictors and 19 PCs resulting 18 candidate features for DI-VNN. | | | |
|  | • | We developed four models with different approaches. First, we applied the simplest model using a logistic regression (LR) with a shrinkage method as recommended by the PROBAST guidelines. | | | |
|  | • | The second and third prediction models were developed using RF and gradient boosting machine (GBM) algorithms. | | | |
|  | • | The last prediction model was developed using the deep-insight visible neural network (DI-VNN) algorithm. | | | |
| *Describe whether and how the model was validated, either internally (e.g. bootstrapping, cross validation, random split sample) or externally (e.g. temporal validation, geographical validation, different setting, different type of participants):* | | | | | |
|  | • | We used participants with ethnicity not from Sulawesi Island for the external validation set. This may demonstrate the model robustness to predict outcomes in the general population. We also randomly split the remaining set after excluding the external validation set. This provided another external validation set with as much as ~20% of the remaining set. For the first to third models, we applied 10-fold cross-validation for hyperparameter tuning and 30 times bootstrapping for training the model using the best hyperparameters. We also applied 10-fold cross-validation to compute the rotated matrix of PCs. For the fourth model, we applied a hold-out cross-validation with 80:20 ratios for the training and validation sets. To compare this model against the others, we applied 30 times bootstrapping to compute the predictive performance. | | | |
| *Describe the performance measures of the model, e.g. (re)calibration, discrimination, (re)classification, net benefit, and whether they were adjusted for optimism:* | | | | | |
|  | • | We used the area under receiver operating characteristics (ROC) curve (AUROC) as the main evaluation metric. This is because the AUROC is threshold-agnostic. But, before evaluating this, we reported the calibration metric of a model using an LR in which the predicted probability as the model output became the only covariate. The models were considered well calibrated if the 95% CIs of the intercept and slope respectively covered 0 and 1, with the probability plots visually aligned with the reference line. | | | |
|  | • | To compare this model against the others, we applied 30 times bootstrapping to compute the predictive performance. To re-calibrate all models using a logistic regression, we also applied 30 times bootstrapping. | | | |
| *Describe any participants who were excluded from the analysis:* | | | | | |
|  | • | (No exclusion) | | | |
| *Describe missing data on predictors and outcomes as well as methods used for missing data:* | | | | | |
|  | • | We checked for missing values in the dataset. The only missing value was found in visual problems for one participant (n=1/1381, 0.072%). This was missing completely at random since we got this information from routine physical health check data. We imputed the missing value using multiple imputation by the chain equation method62 after data transformation using only data in the same data partition. | | | |
|  | |  | | **Dev** | **Val** |
| 4.1 Were there a reasonable number of participants with the outcome? | | | | PY | Y |
| 4.2 Were continuous and categorical predictors handled appropriately? | | | | Y | Y |
| 4.3 Were all enrolled participants included in the analysis? | | | | Y | Y |
| 4.4 Were participants with missing data handled appropriately? | | | | Y | Y |
| 4.5 Was selection of predictors based on univariable analysis avoided? | | | | Y |  |
| 4.6 Were complexities in the data (e.g. censoring, competing risks, sampling of controls) accounted for appropriately? | | | | Y | Y |
| 4.7 Were relevant model performance measures evaluated appropriately? | | | | Y | Y |
| 4.8 Were model overfitting and optimism in model performance accounted for? | | | | Y |  |
| 4.9 Do predictors and their assigned weights in the final model correspond to the results from multivariable analysis? | | | | Y |  |
| **Risk of bias introduced by the analysis** | | | **RISK:** | Low | Low |
|  |  |  | *(low/ high/ unclear)* |  |  |
| *Rationale of bias rating:* | | | | | |
|  | ❖ | We prepared the training set with 20 EPV for LR, 56 EPV for SPC-RF and SPC-GBM, and 21 EPV for DI-VNN. There is no clear consensus yet for standard EPV of DI-VNN, but the predictive performances were similar between internal and external validation sets. For validation, total of the lower outcome in external validation sets were 159 negatives. | | | |
|  | ❖ | No categorization was applied for numerical predictors. | | | |
|  | ❖ | No exclusion was conducted. | | | |
|  | ❖ | Missing data was only 1 instance for a predictor and handled by multiple imputation. | | | |
|  | ❖ | Predictor selection was not conducted by a univariate analysis, but, by principal component analysis, wrapper method, and differential analysis. | | | |
|  | ❖ | Models were evaluated by AUROC and calibration metrics and plot with 30-time bootstrapping for internal and external validation sets. | | | |
|  | ❖ | External validation was conducted using both non-random and random selection. Resampling methods were also applied for internal validation. | | | |
|  | ❖ | All predictors in the final model were part of the multivariable analyses. The weight assessment were not applicable for our modelling approach. | | | |
|  | |  | | | |
| **Overall judgement about risk of bias of the prediction model evaluation** | | | | | |
| **Overall judgement of risk of bias** | | | **RISK:** |  | |
|  |  |  | *(low/ high/ unclear)* |  |  |
| *Summary of sources of potential bias:* | | | | | |
| – | | | | | |

# Table S3. Clinical checklists for assessing suitability of machine learning applications in healthcare

| **Item** | | **Response** | |
| --- | --- | --- | --- |
| Q1. | What is the purpose and context of the algorithm? | ❖ | We developed a questionnaire-free method to predict GDS-15 among community-dwelling elders by machine learning. |
|  |  | ❖ | Patients aged 60 years or older in community-health centre (or equivalent) are eligible. A patient is unlikely well-predicted if the patient input data are not provided by the input options in this application. The exception is religion due to ethical reason, but, religious believers are likely well-predicted, particularly two of the religion options. |
|  |  | ❖ | This application only uses demographics and physical health to predict GDS-15, that avoids frequently asking an elderly patient the same questions. During regular health visit (e.g. monthly), a healthcare giver may input the existing routine information to this application. Only if the GDS-15 is predicted positive, then the questionnaire is given to confirm further diagnostic procedures. |
| Q2. | How good were the data used to train the algorithm? |  |  |
| Q2a. | To what extent were the data accurate and free of bias? | ❖ | We utilized dataset using cross-sectional design from 15 community health centres (CHCs) in Kendari, Indonesia (*n*=1,381). |
|  |  | ❖ | By this design, we can expect similar situation with real-world setting in which this prediction is applied. |
|  |  | ❖ | The selection criteria were all patients aged 60 years or older who visited CHCs with clear consciousness |
|  |  | ❖ | Data were collected from 1,252 patients among which we used some patients for validating our prediction twice (*n*=250 and *n*=129). |
|  |  | ❖ | We balanced the number of positives and negatives to compute the prediction accuracy. There was only 1 missing data of a predictor in data we used to develop our application. This was imputed appropriately using multiple imputation. |
| Q2b. | Were data labelled correctly? | ❖ | Our algorithms were intended to predict GDS-15 results. The prediction is not intended to diagnose major depression disorders. Thus, the data were labelled correctly. |
| Q2c. | Were the data standardized and interoperable? | ❖ | Our prediction can be used in primary care with low-resource setting. The income input is standardized by Big Mac Index; thus, a user just need to choose the country and income using their own currency. There is no need to modify existing electronic medical record. Public can use our prediction via a web application. |
| Q3. | Were there sufficient data to train the algorithm? | ❖ | We prepared the training set with 56 events per variable (EPV) for SPC-GBM and 21 EPV for DI-VNN. While the EPV is sufficient for SPC-GBM (>50 EPV), there is no clear consensus yet for standard EPV of DI-VNN, but the predictive performances were similar between internal and external validation sets. The EPV eligibility is needed to ensure the prediction have a sufficient generalization ability which is a good agreement between internal and external validation. For validation, total of the lower outcome in external validation sets were 159 negatives. This is considerably sufficient (>100). |
| Q4. | How well does the algorithm perform? | ❖ | Our prediction has achieved area under receiver operating characteristics curve (AUROC) of 0.998 (95% CI 0.998 to 0.998; *n*=1,252) in interval validation set. Using the same validation technique, a previous study also applied a questionnaire-free method to predict GDS-15 in older adults living alone by a wearable device. Our prediction outperformed that method (AUROC 0.96, 95% CI 0.91 to 0.99; *n*=47). The previous study did not conduct external validation. |
|  |  | ❖ | We also conducted out-of-sample or external validation. The AUROC is 0.619 (0.610 to 0.627; *n*=129). External validation is intended to show the robustness by giving a stress test, but, it cannot be used for generalization considering the smaller size. This shows AUROC >0.5, which is better than simply guessing. For advanced individual exploration of either the protective or risk factors, we provided a secondary method with similar AUROC 0.579 (95% CI 0.576 to 0.581; *n*=129) using the same data used by the primary prediction method. This is because deep exploration cannot be done using the primary method. |
|  |  | ❖ | We recommend 0.95 for initial threshold while determining another threshold based on local data. This threshold was chosen to pursue sensitivity of 0.9 (95% CI 0.897 to 0.903) in data we used to develop our prediction method. This means only ~10% of GDS-15 positives were falsely predicted as negatives. By this threshold, our method achieved specificity of 0.51 (95% CI 0.504 to 0.517) and 0.607 (95% CI 0.599 to 0.616) in two datasets we used to validate our prediction method. This means ~40 to ~50% of GDS-15 negatives were falsely predicted as positives, leading to more frequent GDS-15 assessment. We cannot compare to other methods because no previous questionnaire-free method to predict GDS-15 has been externally validated. |
|  |  | ❖ | An open-access web application is provided on a public repository (https://predme.app/pre_gds15) to use our algorithms. We invite any investigators to independently validate our algorithms using their own data. |
| Q5. | Is the algorithm transferable to new clinical settings? | ❖ | By the web application, a healthcare giver can recalibrate our prediction using local data to choose a threshold that maximizes the performances in local setting. While finding the best threshold, a healthcare giver can estimate the predictive performances at population level given a threshold using our data. |
| Q6. | Are the outputs of the algorithm clinically intelligible? | ❖ | We provided a secondary method that allows a healthcare giver to critically appraise how the method ends up with the prediction. The algorithm we used for the secondary method enables for achieving such purpose. A healthcare giver can identify the nodes in the network, that include either the protective or risk factors and see these on the saliency maps. Therefore, a healthcare giver knows to what extend our method taking each factor for shifting its decision closer to GDS-15 positive or negative. |
| Q7. | How will this algorithm fit into and complement current workflows? | ❖ | Our prediction can be applied in a monthly basis using a web application we provided for free use. Only if predicted, an older adult is screened by GDS-15. |
|  |  | ❖ | Any healthcare giver can use our web application applying the recommended threshold. |
|  |  | ❖ | No significant change may happen on the existing clinical workflow. The input information is determined by a healthcare giver who meets the patient in-person. |
|  |  | ❖ | Adjusting the threshold based on local data is recommended. This may need a special competence or training. |
| Q8. | Has use of the algorithm been shown to improve patient care and outcomes? | ❖ | We call a pilot study, a prospective clinical trial, and a clinical impact study (effectiveness and cost-effectiveness) for our prediction method. Future investigators can simply use an open-access web application we provided. We expect our method with GDS-15 in a monthly basis to improve early detection of major depression disorders under <1 year compared to annual GDS-15. But, such intervention may need further investigations. |
| Q9. | Could the algorithm cause patient harm? | ❖ | The false negative result may cause depressive symptoms unscreened. To prevent the harms, a user need local data to improve the predictive performance. A clinician should also continuously questioning counterintuitive internal properties of a prediction by our algorithms. |
| Q10. | Does the algorithm raise ethical, legal or social concerns? | ❖ | We consider ethical, legal, and social issues when developing a web application to apply our prediction. |
|  |  | ❖ | Religion options is provided for many religions to keep the application inclusive and avoid inequality. We also used Big Mac index which is commonly used to convert income to the same notion in any country; thus, we can expect its application to any country. |
|  |  | ❖ | Our algorithms are also equitable and inclusive because of no need of high resources, including those for the electronic medical record system. |
|  |  | ❖ | Apart of availability of an independent validation and a clinical trial for our prediction method, a local recalibration is warranted, because a perfect consistency of predictive performances is difficult if not impossible. A responsibility to ensure the recalibration is on the healthcare provider. A competent staff for adjusting the local or individual threshold (i.e. a clinical informatician, a clinical pathologist, an existing biostatistician, or a trained physician) is also needed. Failed to provide both efforts may raise legal issues using our method. |
|  |  | ❖ | Non-commercial use is permitted. Local recalibration and a competent is preferred before our method is commercialized in a healthcare provider. Any commercialization need to apply permission to the developer of this algorithm. |
|  |  | ❖ | Social issue may be raised if the commercialization is applied, especially in non-insured healthcare service. Anyway, this prediction is not worthy to put the cost on the patient. |

# Table S4. The 15-item Geriatric Depression Scale (GDS-15) questionnaire

| **ENGLISH - GERIATRICS DEPRESSION SCALE (SHORT-FORM)** | | | |
| --- | --- | --- | --- |
| Instruction: Please put a tick "√" in the box "□" next to the answer of your choice based on your felt over the past week | | | |
| A. Yes: if you feel, and experience; B. No: if you don't feel, and experience | | | |
|  |  | Yes | No |
| 1 | Are you basically satisfied with your life? | □ | □ |
| 2 | Have you dropped many of your activities and interest? | □ | □ |
| 3 | Do you feel that your life is empty? | □ | □ |
| 4 | Do you often get bored? | □ | □ |
| 5 | Are you in good spirits most of the time? | □ | □ |
| 6 | Are you afraid that something bad is going happen to you? | □ | □ |
| 7 | Do you feel happy most of the time? | □ | □ |
| 8 | Do you often feel helpless? | □ | □ |
| 9 | Do you prefer to stay in your room, rather than going out and doing new things? | □ | □ |
| 10 | Do you feel you have more problems with memory than most people? | □ | □ |
| 11 | Do you think it is wonderful to be alive? | □ | □ |
| 12 | Do you feel pretty worthless the way you are now? | □ | □ |
| 13 | Do you feel of energy? | □ | □ |
| 14 | Do you feel that your situation is hopeless? | □ | □ |
| 15 | Do you think that most people are better than you are? | □ | □ |
|  | Thank you very much for your cooperation. |  |  |
|  | | | |
| **BAHASA INDONESIA - SKALA DEPRESI LANSIA (FORM-SINGKAT)** | | | |
| Perintah: Harap beri tanda "√" di kotak "□" di sebelah jawaban anda. Pilih sesuai yang anda rasakan seminggu terakhir. | | | |
| A. Ya: jika anda merasakan, dan mengalami; B. Tidak: jika anda tidak merasakan, dan mengalami | | | |
|  |  | Ya | Tidak |
| 1 | Apa anda merasa puas dengan hidup anda? | □ | □ |
| 2 | Apakah anda meninggalkan banyak kegiatan dan hobi anda? | □ | □ |
| 3 | Apakah anda merasa hidup anda tidak berharga? | □ | □ |
| 4 | Apakah anda sering merasa bosan? | □ | □ |
| 5 | Apakah anda selalu bersemangat ketika melakukan kegiatan? | □ | □ |
| 6 | Apakah anda berfikir sesuatu yang buruk akan menimpa anda? | □ | □ |
| 7 | Apakah setiap saat anda selalu bahagia? | □ | □ |
| 8 | Apakah anda sering merasa tidak berdaya? | □ | □ |
| 9 | Apakah anda lebih senang di kamar dibandingkan pergi keluar dan mengerjakan hal-hal baru? | □ | □ |
| 10 | Apakah anda merasa anda lebih sulit mengingat dibandingkan orang lain? | □ | □ |
| 11 | Apakah anda fikir kehidupan anda patut disyukuri? | □ | □ |
| 12 | Apakah anda merasa anda tidak berguna? | □ | □ |
| 13 | Apakah anda merasa sangat bersemangat? | □ | □ |
| 14 | Apakah anda merasa anda dalam kondisi tidak memiliki harapan? | □ | □ |
| 15 | Apakah anda fikir orang lain lebih baik dari anda? | □ | □ |
|  | Terima kasih banyak atas kerjasamanya. |  |  |

# Table S5. Outcome-leakage handling

| **Procedure** | **Outcome-leakage handling** |
| --- | --- |
| Data collection | A participant answered the questions with assistance of a trained assessor. The GDS questionnaire is described in Table S4. The trained assessor that assisted a participant to fill out the GDS questionnaire was also blinded to the predictor information. Predictor data were demographic data and routine physical health check results from other healthcare givers without knowing the assessment results of depressive symptoms. |
| Standardization and normalization of numerical predictors | We only used the mean and SD calculated from data partitioning for model development. Numerical predictors in any data partitions were standardized using these values. |
| Missing value imputation | We imputed the missing value using multiple imputation by the chain equation method (Caramelli et al.) after data transformation using only data in the same data partition. Randomly, the missing value was a part of data partitioning for model development. |
| Predictor extraction, representation, and selection | We only used data partitioning for model development to conduct predictor extraction, representation, and selection.  A ten-fold cross-validation procedure was applied on only data partitioning for model development. We used average values computed from ten rotated matrices of PCs to represent 37 binarized and numerical predictors into 19 PCs. We also used average values of data partitioning for model development to get those PCs for model validation. Details on a resampled dimensional reduction method for this study was already described elsewhere (Sufriyana et al., 2021). |
