# Supplementary material for: Questionnaire-free machine-learning method to predict depressive symptoms among community-dwelling older adults
Source: PLoS One. 2023 Jan 25;18(1):e0280330. doi: 10.1371/journal.pone.0280330 (PMC9876369; doi:10.1371/journal.pone.0280330)
Supplement: Attachment — Submitted filename: rebuttal_letter.docx [file pone.0280330.s003.docx]

################################################################################

######## 1 ######## Academic editor

################################################################################

###### 1.1 ###### Based on the reviewers' comments, you are asked to provide a revised version of the manuscript addressing all their concerns.

Response:

Thank you for giving us one more chance to revise the manuscript. We have addressed all the reviewers’ comments. The revision is tracked in the manuscript and some are either commented in the manuscript or shown in this rebuttal letter.

################################################################################

######## 2 ######## Reviewer 1

################################################################################

Thank you to the authors for responding to my comments. Many of the edits were very helpful, but I have further recommendations that I believe could strengthen the manuscript. I also recommend having the manuscript revised for language clarity prior to the next submission. I’ve pointed out some specific examples regarding language clarity in my comments, but it would be difficult to list all of them. I recommend taking another general pass, or potentially asking an external scientific writer to help revise the manuscript. Some of these language issues are grammatical (eg, see my updated comment 2.1.2), but additionally about the specific word choices that justify the conclusions based upon the results or extracted from cited references (eg, see my updated comment 2.2.1).

My points are numbered by the numbering used by the authors in their point-by-point responses. Thank you.

Response:

Thank you for appreciating our previous revision. We agree to take another general pass with an external writing assistance. The revision is tracked all over the manuscript.

###### 2.1 ###### Major comments
                  #### 2.1.2 #### I thank the authors for confirming that data leakage did not occur. I think the language could be made clearer. I believe the authors mean by “data partitioning” that within each cross-validation split, all standardization and MICE parameters were computed within the training data, and applied to both the training and validation data. Please confirm, and if true, clean up the language in the methods. “Data partitioned for model development” for example, is clearer language with the appropriate tense compared to “data partitioning for model development”.

Response:

Thank you for making the language clearer. What we mean by “data partitioning” was a subset after partitioning data for either development or validation. We agree that the language was unclear; thus, changes are made according to your suggestion. The revision is tracked and commented in the manuscript but not shown below.

#### 2.1.3 #### Thank you for clarifying the intention of the external validation set in the paper. I recommend adding an extra sentence in the Limitations section stating that despite the reliability of the model demonstrated in the paper using external validation, one should still not assume generalizability. More testing is needed.

Response:

Thank you for this suggestion. This statement is correct to some extent. We agree to add it in the limitations. The revision is tracked and commented in the manuscript, and also shown below.

Revision:

Eventually, despite the model’s reliability demonstrated in the paper using external validation, one should still refrain from assuming generalizability for any other population with different characteristics. External validation is still required for such a population, yet, this is a general issue in prediction studies, not limited to our study.

#### 2.1.4 #### I understand the clarification between prediction models and risk identification. I am also not sure of the relevance of multivariate versus multivariate models the authors are making in the paper, and would recommend its removal. On the former point regarding survey fatigue, I am not sure I agree that the authors proposed system is less arduous than depression screening. The authors’ proposed system requires the collection of clinical data (eg, on commorbidities, health conditions, hearing problems), which would require some amount of interaction with the healthcare system. One could argue that accessing annual clinician check-ins is as arduous as self-reported surveys, or a clinical depression assessment. I also am not sure the point on “untruthful or inconsistent responses” in the Introduction is a good argument, given you’re still validating a model against a yes/no self-report of depression symptoms - something that by the authors' argument could be an “untruthful” assessment as ground truth - and thus the model would just propagate this response bias on a large scale. This is analogous to how prediction models propagate bias in data broadly, for example see, https://doi.org/10.1073/pnas.2204529119. I would suggest removing this point as well. That being said, I think the point in the introduction on the developed model acting as an EHR indicator to triage patients for a mental health follow-up is sufficient for justifying the research, and the discussion paragraph regarding “Notably, we conducted this study [...]” can be removed.

Response:

Thank you for your clarification to the previous comment for this issue. We agree to remove this paragraph under section Discussion; thus, neither tracked changes nor comment are shown in the manuscript. Also thank you for appreciating our point in the Introduction. We also changed it according to comment 2.2.2 below.

###### 2.2 ###### Minor comments
                  #### 2.2.1 #### I recommend changing the language to not state so objectively the accuracy of the GDS-15, but instead state that a “of which a recent systematic review and meta-analysis found that the 15-item version (GDS-15) is the most accurate”, since the authors are basing this point on a single paper.

Response:

Thank you for asking the language to not state so objectively the accuracy of the GDS-15. We agree to modify it according to your suggestion. Indeed, this point is based on a single paper but not a single study. Fortunately, this systematic review and meta-analysis provided us a better lens to GDS-15 than if we just arbitrarily cited several papers, which unlikely covered the previous studies in a comprehensive way. The revision is tracked and commented in the manuscript, and also shown below.

Revision:

To screen depressive symptoms in older adults, the Geriatric Depression Scale (GDS) is one of the most commonly used questionnaires. A recent systematic review and meta-analysis found that the 15-item version (GDS-15) is the most accurate compared to the shorter or longer versions (Krishnamoorthy et al., 2020).

Reference:

\##### Krishnamoorthy, Y., Rajaa, S., and Rehman, T. (2020). Diagnostic accuracy of various forms of geriatric depression scale for screening of depression among older adults: Systematic review and meta-analysis. Arch Gerontol Geriatr 87, 104002. 10.1016/j.archger.2019.104002.

#### 2.2.2 #### I recommend removing the statement “questions asked later in the long-term”, and as suggested in my response to 2.1.4, I do not think response bias is a good motivation for this prediction model. I recommend removing this section.

Response:

We really appreciate this suggestion. The statement was removed and substituted with one focusing on the model as an EHR indicator. The revision is tracked and commented in the manuscript, and also shown below.

Revision:

By questionnaire-free variables, demographic and physical health data from routine visits can be utilized as an electronic health record (EHR) indicator to triage patients for a mental health follow-up by GDS-15.

#### 2.2.4 #### I understand the authors’ point regarding the preference for machine learning. I recommend stating it explicitly upfront in the introduction, clarifying the word "insufficient" within the same sentence. For example: “[...] using logistic regression (LR) may be insufficient because its simplicity does not accurately reflect the complexity of real-world data [citation justifying this conclusion]. Machine learning [...]”.

Response:

Thank you for your suggestion. However, we disagree to explain the insufficiency due to its simplicity uncovering the real-world complexity. Such view is considerably disrespectful in another field which used regression algorithms in the majority of prediction studies, if not all. We tried to avoid dichotomy between LR and non-LR, and focus on problem-centric instead of method-centric solutions. In this study, as described in the main text, we tried to enlarge our hypothesis space of an accurate model of GDS-15 prediction, not limited to a probability theory. No revision is shown in the manuscript.

################################################################################

######## 3 ######## Reviewer 3

################################################################################

I can see that authors have incorporated most of the changes as asked by the reviewers.

Response:

Thank you for confirming our revision to the previous reviewer’s comments. Here below we described more revisions according to your comments.

###### 3.1 ###### Novelty of the proposed approach is either missing or the authors fail to express it in the manuscript. Novelty should be explained.

Response:

Thank you for pointing out this issue. We have already described the novelty, but we also believe it is failed to stand out in the manuscript. Since this is not a methodological paper, our novelty description focuses on the application novelty. To signify the novelty description, we added sub-sections in the Introduction. The novelty is described in sub-section “Previous works”. The revision is tracked and commented in the manuscript, and also shown below.

Revision:

**Previous works**

Almost all existing predictive models of depressive symptoms include questionnaire-based predictors, e.g., the Patient Health Questionnaire (PHQ), the Edinburgh Postnatal Depression Scale (EPDS), and the GDS (Smithson and Pignone, 2017). More-frequent identification of patients with depression (with an area under the receiver operating characteristic [ROC] curve [AUROC] of 0.700, 95% CI 0.629 to 0.771) still needed several questionnaire-based screening tools (Chekroud et al., 2016). They were a part of the Self-Reported Quick Inventory of Depressive Symptomatology (QIDS-SR) and Hamilton Depression Rating Scale (HAM-D). A previous study developed an extended predictD algorithm to predict major depression 12~24 months later based on the DSM-IV (AUROC 0.728, 95% CI 0.675 to 0.781; n=2670), but this also needs a subject to fill in the 12-Item Short Form (SF-12) for two of the predictors (King et al., 2013).

One study utilized a wearable device to predict GDS-15 and HAM-D results in older adults (AUROC 0.96, 95% CI 0.91 to 0.99; n=47) (Kim et al., 2019). Unfortunately, the sample size was small, and a wearable device might not be affordable for some older adults. However, no previous study developed a questionnaire-free method to predict depressive symptoms based on standard screening questionnaires in community-dwelling older adults.

References:

\##### Chekroud, A.M., Zotti, R.J., Shehzad, Z., Gueorguieva, R., Johnson, M.K., Trivedi, M.H., Cannon, T.D., Krystal, J.H., and Corlett, P.R. (2016). Cross-trial prediction of treatment outcome in depression: a machine learning approach. Lancet Psychiatry 3, 243-250. 10.1016/s2215-0366(15)00471-x.

\##### Kim, H., Lee, S., Lee, S., Hong, S., Kang, H., and Kim, N. (2019). Depression Prediction by Using Ecological Momentary Assessment, Actiwatch Data, and Machine Learning: Observational Study on Older Adults Living Alone. JMIR Mhealth Uhealth 7, e14149. 10.2196/14149.

\##### King, M., Bottomley, C., Bellón-Saameño, J., Torres-Gonzalez, F., Svab, I., Rotar, D., Xavier, M., and Nazareth, I. (2013). Predicting onset of major depression in general practice attendees in Europe: extending the application of the predictD risk algorithm from 12 to 24 months. Psychol Med 43, 1929-1939. 10.1017/s0033291712002693.

\##### Smithson, S., and Pignone, M.P. (2017). Screening Adults for Depression in Primary Care. Med Clin North Am 101, 807-821. 10.1016/j.mcna.2017.03.010.

###### 3.2 ###### Authors should explain the contributions made in the paper by adding a sub-section in the first section along with a sub-section on motivation.

Response:

Thank you for this suggestion. We have added several sub-sections in the first section, including a sub-section on “Motivation”. We also explained our contribution in sub-sections “Previous works” and “Intuition”. The revision is tracked and commented in the manuscript, and the description on contribution is also shown below.

Revision:

However, no previous study developed a questionnaire-free method to predict depressive symptoms based on standard screening questionnaires in community-dwelling older adults.

**Intuition**

Later-life (aged 60+) depression is associated with several factors, and their assessments can utilize routine databases at the first visit of a subject to a healthcare facility.

###### 3.3 ###### Paper requires a flowchart which can show the flow and step by step approach of the proposed methodology in tackling the undertaken problem.

Response:

Thank you for this suggestion. We have added the flowchart as Figure 1. The revision is tracked and commented in the manuscript, and also shown below.

**Figure 1. Flowchart of the proposed methodology.**

###### 3.4 ###### Formatting mistakes are there; such as random forest [RF] , here it should be written as random forest (RF), Similar errors should be removed.

Response:

Thank you for your suggestion. Previously, we used square brackets for bracket nesting to avoid using parentheses inside parentheses, in order to keep formatting consistently in US English. We have rechecked and did not find if we used square brackets outside parentheses. No revision is made for this comment.
